# Linguistic Validation and Reliability of the Croatian Version of the TOPICOP Questionnaire

**DOI:** 10.3390/medicina60060968

**Published:** 2024-06-12

**Authors:** Adela Markota Čagalj, Josko Markic, Dubravka Vuković, Zdenka Šitum Čeprnja, Tina Gogić Salapić, Ivan Buljan, Shelly Melissa Pranić

**Affiliations:** 1 Department of Dermatology and Venereology, University Hospital of Split, Spinčićeva 1, 21000 Split, Croatia; adela.markota@gmail.com (A.M.Č.); zdenkasitum7@gmail.com (Z.Š.Č.); tina.gogic1@gmail.com (T.G.S.); 2 School of Medicine, University of Split, Šoltanska 2, 21000 Split, Croatia; jmarkic@mefst.hr; 3 Department of Pediatrics, University Hospital of Split, Spinčićeva 1, 21000 Split, Croatia; 4 Department of Psychology, Faculty of Humanities and Social Sciences in Split, University of Split, Poljička cesta 35, 21000 Split, Croatia; ibuljan@ffst.hr; 5 Department of Public Health, School of Medicine, University of Split, Cohrane Croatia, Šoltanska 2, 21000 Split, Croatia; spranic@mefst.hr

**Keywords:** TOPICOP questionnaire, atopic dermatitis, allergic contact dermatitis, children, adults

## Abstract

*Background and Objectives*: This study demonstrates the factor structure and reliability of the Croatian version of the TOPICOP (Topical Corticosteroid Phobia) questionnaire, thereby contributing to further validation and standardization of the measurement of topical corticophobia with dermatological patients or their parents, which directly affects patient or parent compliance, as well as the final therapeutic effect. *Materials and Methods*: The cross-sectional, observational study was conducted at the University Hospital Centre Split, Department of Dermatovenerology. The research involved inviting 120 participants (age 12–68) who attended the University Hospital Centre Split’s Atopy School, patients examined in the Dermatology Outpatient Clinic of the University Hospital Centre Split and diagnosed with atopic dermatitis (AD) or allergic contact dermatitis (ACD), and parents or legal representatives of patients younger than 12 years old. The TOPICOP questionnaire consists of 12 items assessing the three different components of topical corticophobia (worries, beliefs, and behaviour). Respondents evaluated their perception of the correctness of each statement within 10 min of filling out the questionnaire on a four-point Likert scale. *Results*: The response rate was 94%, resulting in a sample of 113 respondents (109 adults and 4 children). Factor analysis yielded one common factor of relatively high reliability (Cronbach α = 0.85, 95% CI 0.81 to 0.89). No differences were found in questionnaire scores between male and female participants, nor between the parents/legal representatives of dermatological patients and other patients. *Conclusions*: This research contributes to further development of the appropriate measuring instrument, its practical application, and thus, the better recognition, resolution, and prevention of topical corticophobia as part of the better overall healthcare and treatment of chronic dermatological patients.

## 1. Introduction

Atopic dermatitis (AD) is a chronic, recurrent, inflammatory skin disease characterized by continuous itching of the skin [1]. Worldwide, AD affects about 101.27 million adults and 102.78 million children, translating to prevalence rates of 2.0% (95% confidence interval from 1.4 to 2.6) and 4.0% (95% confidence interval from 2.8 to 5.3), respectively [2]. Therefore, it occurs predominantly in the pediatric population and is common among children between 0 and 5 years old, affecting around 20% of them [3,4]. AD is caused by a combination of pathophysiological factors, such as genetic disorders, a defect in the epidermal barrier, an altered immune response, and disruption of the skin’s microbiome [1]. Acute flare-ups of eczematous, oozing, and pruritic lesions over dry skin are hallmarks of AD. Chronic lesions include prurigo nodules and reddish-brownish patches of dry, cracked, or scaly skin with lichenification. Itchy skin, particularly at night, causes weariness, disturbed sleep, and mental health issues. Clinical manifestations, as well as the patient’s and family’s medical history, are taken into account while making a diagnosis. Both objective and subjective signs and symptoms must be evaluated in order to identify the overall severity of the condition [5]. In terms of care, it is critical to avoid trigger factors, maintain skin hydration, and manage skin inflammation. The control of skin inflammation is achieved with the application of topical corticosteroids (TCSs) or immune modulators. Family involvement in the treatment process and its associated high costs substantially reduce the quality of life of patients and their families [3,5].

Another common inflammatory skin condition brought on by exposure to an environmental factor is allergic contact dermatitis (ACD), which can manifest as acute, subacute, or chronic dermatitis [6]. Some research papers demonstrate prevalence rates as high as 20% of the general population [7]. The first phase of the type IV delayed-type hypersensitivity reaction is sensitization, which occurs when a person is first exposed to an allergen. The second elicitation phase takes place after re-exposure to the antigen. The recruitment and activation of antigen-specific effector memory T cells in the skin lead to skin inflammation in sensitized individuals [8]. Clinical manifestations often include bullae, vesiculation, scaling, and erythema in the acute phase, and lichenification and fissuring in the chronic phase. The dermatitis is typically restricted to the place of contact; however, patchy or diffuse disease can also occur [9]. Although patch testing is the gold standard for diagnosing ACD, determining hidden allergy sources requires careful linkage between the history and physical examination [10]. The most important aspect of ACD treatment is the avoidance of the responsible allergen, but as in AD, TCSs are the mainstay of treatment when it comes to controlling inflammation [6].

Increased allergen penetration, immunological dysregulation, including common cytokine pathways, the frequent use of emollients and topical medicines, and other characteristics, may predispose patients with AD to developing ACD. According to recent systematic reviews, ACD poses a serious clinical issue for AD patients of all ages [11].

TCSs are an effective and safe treatment used in the treatment of AD and ACD when administered under medical supervision. The strategy can be either proactive or reactive, depending on the severity of the condition. In patients with moderate to severe illness, proactive therapy with TCSs or topical calcineurin inhibitors is much better than reactive therapy at lowering the frequency of flares and lengthening the time between them [12]. Potency and potential side effects categorize TCSs [13]. The most common adverse effects of TCSs include striae, atrophy, perioral dermatitis, acneiform eruption, rosacea, and purpura. Additional skin-related side effects include telangiectasias and erythema, gluteal granulomas, folliculitis, periocular dermatitis, delayed wound healing, hypopigmentation, hypertrichosis, secondary infection, the worsening of dermatophyte infection (tinea incognito), and contact dermatitis. Using TCSs can increase the risk of mucocutaneous infections. TCSs’ ability to decrease inflammation may be responsible for the unusual presentation, development, and duration of cutaneous infections. Although it is uncommon, contact sensitization to TCSs is possible. In the event that contact sensitization is suspected, it is critical to distinguish it from hypersensitivity to other topical therapy ingredients. TCS use has been linked to systemic adverse effects such as Cushing syndrome, cataracts, glaucoma, adrenal suppression, reduced growth rate, hypertension, and hyperglycemia. Children’s higher body surface area to weight ratio makes them more vulnerable to systemic negative effects. TCSs with low potency rarely cause adverse effects at all. Even with super-high-potency formulations, the evidence suggests that the danger of systemic adverse effects in humans is low if doses do not exceed 50 g per week. However, higher potency, prolonged usage, occlusion, and application to a greater area or to places with thinner skin, such as the face and genitalia, enhance the risk of all the listed adverse effects. Lower potencies and shorter periods of use are recommended for children [14]. The correct application of TCSs using the fingertip unit method is essential for successful use [13]. A fingertip unit is the quantity of medication applied from the tip of the index finger to the distal skin crease, as expressed from a tube with a 5 mm diameter nozzle. It covers approximately 2% of an adult’s body surface area [15].

Nonadherence to prescribed TCSs is often a result of phobia secondary to false information. The fear of using TCS therapy is called topical corticophobia. This complex, multidimensional phenomenon involves incorrect perceptions and vague negative feelings that patients and their parents have concerning TCSs, thus interfering with the successful treatment of dermatologic disorders [3]. They frequently worry about the side effects of TCSs and potential rebound effects after they stop using them [16]. One systematic review of the literature found that among AD patients, the prevalence of topical corticophobia ranged from 21.0% to 83.7% [17]. As anticipated, in studies comparing nonadherence to TCS therapy between patients in corticophobic and non-corticophobic groups, corticophobics demonstrated considerably greater rates of nonadherence to topical therapy with TCSs than non-corticophobics [18,19]. The Internet and social media are the main sources of misinformation regarding TCS treatment [20]. The potential negative consequences, such as thinned skin and stunted growth, are frequently overstated. Additionally, multiple websites promote tests or consultations to identify the ‘cause of allergy’, and ‘natural’ creams as alternative treatments, which can sometimes contain hidden notable quantities of TCSs [20]. Other possible causative factors for topical corticophobia include personally experienced side effects, a higher number of general practitioner visits, polypharmacy, frequent changing of dermatology clinics, a lack of education about TCSs, and conflicting advice from family members, friends, and medical professionals [21]. Therefore, dermatologists should be vigilant against false information about TCSs and prepared to provide their patients with detailed explanations grounded in solid data.

Another crucial component of effective treatment for chronic dermatological diseases, such as AD or ACD, is education of the patients and parents in the pediatric population [22]. We organized educational sessions and workshops in the form of the Atopy School in the University Hospital Centre Split, where patients from all over Dalmatia are referred and treated. Topics such as skin care, AD therapy, corticosteroid concerns, and allergological evaluation were covered. A multidisciplinary team, including pediatric allergologists, psychologists, dermatologists, and nutritionists, led the sessions. A trained nurse held a workshop on the care of patients with AD or ACD. Following the workshop and sessions, parents, caregivers, and medical experts had a discussion on the given topics.

Due to the noted high impact of topical corticophobia on the effective treatment of dermatologic diseases and the high prevalence of AD, in 2013, the TOPICOP (Topical Corticosteroid Phobia) scale was developed and validated to evaluate patients or their parent’s steroid phobia. The measurement and comparison of topical corticophobia have been facilitated by the use of this questionnaire. It consists of 12 items, each scored on a four-point Likert scale (0 = totally disagree or never, 1 = not really agree or sometimes, 2 = almost agree or often, and 3 = totally agree or always) [23]. TOPICOP is, at this point, the only score validated to evaluate topical corticophobia. An international feasibility study was conducted in 17 countries around the world in 2017, and questions were considered clear or extremely clear by more than 80% of participants. The items “TCSs pass into the bloodstream”, “TCSs can lead to infections”, and “TCSs can lead to asthma” were the most difficult to answer. This is not surprising, given that conflicting evidence has prevented even medical professionals from reaching a consensus on some of these things [24].

With the aim of creating a platform for assessing topical corticophobia in our patients, we decided to translate the TOPICOP scale to the Croatian language. Participants of the University Hospital Centre Split’s Atopy School, patients examined in the Dermatology Outpatient Clinic of the University Hospital Centre Split and diagnosed with AD or ACD, and the parents or legal representatives of patients younger than 12 years old were asked to complete the Croatian version of the TOPICOP questionnaire. The primary objective of this research was to test the validity and reliability of the Croatian version of the TOPICOP questionnaire. We wanted to assess the degree of steroid phobia in a broad group of dermatological patients at our centre and identify sources from which patients obtain information regarding TCSs, regardless of the class of TCSs they use.

## 2. Materials and Methods

We conducted a cross-sectional study at the University Hospital Centre Split, Croatia, between January and September 2023. Participants of the Atopy School, patients examined and diagnosed with AD or ACD in the Dermatology Outpatient Clinic, and parents or legal representatives of patients younger than 12 years old were invited to complete the Croatian version of the TOPICOP questionnaire. The research involved inviting 120 respondents between the ages of 12 and 68 who voluntarily agreed to the research. We included participants from all over Dalmatia, to make the sample as representative as possible. The objective of the study was to assess the validity and reliability of a translation of the TOPICOP questionnaire into the Croatian language.

TOPICOP is a self-administered scale that contains 12 items related to worries, beliefs, and behaviour [25]. Each item was scored on a 4-point Likert scale (0 = totally disagree or never, 1 = not really agree or sometimes, 2 = almost agree or often, and 3 = totally agree or always). The final result is a summation that ranges from 0 to 36, expressed as a percentage. Higher values are related to more severe topical corticophobia [24,25]. Two bilingual independent translators and experts in dermatology, whose mother tongue was Croatian, translated the questionnaire from English to Croatian. The first translator was knowledgeable about healthcare terminology and the content area of the TOPICOP questionnaire, while the second translator was familiar with colloquial phrases, healthcare slang and jargon, idiomatic expressions, and emotional terms in common use in Croatian. A third bilingual translator, whose mother tongue was English, compared two forward-translated versions of the TOPICOP questionnaire to each other and to the original version to identify ambiguities and discrepancies in words, sentences, and meanings. She carried out a back-translation to English, without access to the original version of the questionnaire. The research team and the three translators reached consensus, resulting in the final Croatian translation of the TOPICOP questionnaire.

The eligibility criteria included fulfilling the Hanifin and Rajka diagnostic standards for AD [26] or having a positive patch test result, as well as a medical history supporting an ACD diagnosis [6], Croatian as the mother tongue, and age 12 and older. The study included the parents or legal representatives of children younger than 12 years old as participants. The non-inclusion criteria were psychiatric comorbidities that would hinder the participant’s understanding of the questionnaire. Exclusion criteria included more than 10% of missing responses in questionnaires. Following the educational sessions and workshop at the Atopy School in March and June 2023, as well as the dermatological examinations conducted in the Dermatology Outpatient Clinic between January and September 2023, we asked patients diagnosed with AD or ACD, as well as the parents or legal representatives of children under 12 diagnosed with AD or ACD, to complete the TOPICOP questionnaire. The researchers informed the participants about the reasons for conducting the research and emphasized the importance of answering correctly. In case some of the respondents needed help filling out the questionnaire or asked for some extra information, the researchers were available at all times. Participants used pens and paper to complete the questionnaire within 10 min. The response rate was 94%, resulting in a sample of 113 respondents (109 adults and 4 children).

In the first step of the analysis, the factor structure of the questionnaire was assessed using factor analysis. After confirmation of the single factor structure, we assessed reliability and descriptive parameters (distribution normality using the Shapiro–Wilk test; the mean and standard deviation were presented for each item and the entire scale).

Due to the discrepancy in sample sizes between groups, sex comparisons and patient status comparisons were performed using the Mann–Whitney U test. The collected data were analyzed using the JASP statistical programme v.0.18.1.0. (JASP Team, 2023).

## 3. Results

### Reliability and Validation of the Questionnaire

All questionnaire items were connected to one common factor (Table 1), which makes the calculation of the total result based on the sum of all items justified. The reliability of the entire questionnaire was good; Cronbach’s alpha coefficient of internal consistency was α = 0.85 (95% confidence interval from 0.81 to 0.89).

When looking at the descriptive statistics of each individual item, participants were the least likely to believe that using corticosteroids could lead to infection and weight gain, but at the same time, they were afraid to apply TCSs to areas with thin skin and in thick layers (Table 2).

The average score on the questionnaire was M = 28.5 (SD = 6.9, min-max 12–42), and the results were normally distributed (Shapiro–Wilk test, *p* = 0.151).

**Figure 1:**
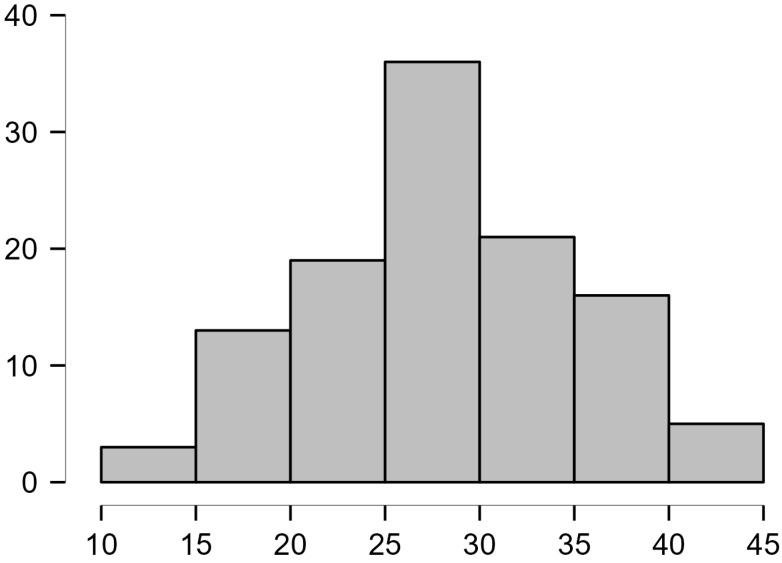
Distribution of participants’ results on the TOPICOP questionnaire (*n* = 113).

The distribution of the results can be seen in Figure 1. No statistically significant differences were found between male *(n* = 16) and female (*n* = 97) participants (Mann–Whitney, *p* = 0.142). There was no difference between dermatological patients (*n* = 37) and others (*n* = 76) (Mann–Whitney, *p* = 0.558), nor between parents of dermatological patients (*n* = 90) and other parents (*n* = 23) (Mann–Whitney, *p* = 0.895).

## 4. Discussion

Even though the pathophysiology of AD has been better understood over the decades, especially with regard to its inflammatory features and the availability of very effective medications such as TCSs for treatment of the condition, many children and adults suffering from AD still struggle to achieve adequate control over their symptoms. The burden of AD on children and adults is extensive, and it also has a notable influence on the lives of family members and patient caregivers. This multifaceted effect has implications for quality of life, occupational productivity, and mental health in affected adults. The effects of AD on children are similar to those of other chronic childhood illnesses such as cystic fibrosis, epilepsy, and cerebral palsy. Children with AD frequently experience everyday struggles, such as difficulties eating, dressing, and playing, which prevents them from having a “normal childhood” [27].

Unfortunately, a common cause of unsuccessful AD management is low adherence to TCS treatment, leading to AD flares. Steroid phobia is widespread and can make it difficult to comply with treatment, which lowers its efficacy. The reporting and measurement of TCS fear have shown a great deal of variation [17]. Nonetheless, since the TOPICOP scale was created relatively recently, it is now feasible to standardize and compare data across various groups. Currently, it is the only validated score that has been used globally for investigating topical corticophobia in AD.

The majority of the “best practice” guidelines for score validation are followed by the statistical validation technique that is described here [28]. Reliability analysis was assessed by Cronbach’s alpha, and a value greater than 0.8 is considered adequate [29]. These findings are consistent with reports from previous studies [25]. Moreover, we calculated construct validity using factor analysis and reliability. Items had a strong communality with the one factor identified from the principal component analysis. The combined TOPICOP score averaged around 28.5, showing no significant difference between male and female participants. In our daily practice, topical corticophobia primarily affects parents; therefore, the majority of the study respondents were adults, including parents of pediatric patients and adult patients. We recruited study participants from hospital outpatient dermatology departments and the Atopy School to minimize potential recruitment bias.

While TOPICOP clearly possesses superior psychometric properties, it is not yet apparent if it can effectively represent the essence of the topical corticophobia idea. Since its initial appearance in relation to asthma 25 years ago, corticophobia has come to be known as a prevalent but poorly understood condition [30]. Given that phobias are defined by psychiatrists as baseless fears that can limit a person’s capacity to communicate, work, or carry out regular activities [31], the term ‘corticophobia’ seems a bit overused in regard to this description. It is also clear that worries about TCSs are not always unfounded. For example, it is true that TCSs can enter the bloodstream and cause irreversible thinning and vasoplegia, which can damage skin. Thus, the important thing to consider is not if a patient’s worries and impressions regarding TCSs are accurate or not, but rather how big of an impact they have on treatment compliance.

Worries, beliefs, and behaviour are related; hence, items from these three dimensions of topical corticophobia in AD are included in the TOPICOP scale. Questionnaire items concerning beliefs (systemic side effects, infections, weight gain, cutaneous side effects, and asthma induction) align with those previously mentioned by several writers [32,33]. Worries and behaviour-related items (TCS dependency or addiction, loss of efficacy, and need for reassurance) have also been documented [33,34]. Furthermore, a significant number of respondents in our study stated that they were hesitant to use TCSs in areas with thin skin and in thick layers, even though they were the least likely to believe that using TCSs could lead to infection and weight gain, demonstrating that unfavourable attitudes and views about the treatment were not necessarily supported by scientific research [35]. Their average TOPICOP scale ratings showed that topical corticophobia is a significant and common issue in AD and ACD treatment, which causes patients or parents of pediatric patients to adhere to prescribed local treatment regimens poorly, resulting in therapeutic failures.

Although physicians regularly question patients about their attitudes toward corticosteroids during consultations, they rarely investigate the causes of these worries. Topical corticophobia may be a consequence of numerous factors, such as personal experience, conflicting advice from friends and family, information from the internet, etc. A recently conducted cross-sectional study in Italy demonstrated that high parental education, older age of patients, early disease onset, and a high parental DLQI are the major risk factors influencing severe parental corticophobia. This is understandable, given that parents’ poor living conditions may also influence their treatment choices [36]. A Danish study revealed the opposite, demonstrating a significant association between a high global TOPICOP score and low parental educational attainment, which causes AD flare-ups to be treated later [37]. Surprisingly, one study showed significant topical corticophobia among health workers, especially pharmacists and general practitioners, which has an additional impact on the patients’ inadequate information [38]. Therefore, the education of patients and parents of pediatric patients, as well as the re-education of healthcare providers, are crucial. A study in the Netherlands demonstrated that interactive digital education for healthcare providers is an efficient tool to attain prolonged improvements in knowledge on TCSs and corticophobia [39]. Physicians or medical organizations may consider expanding their online presence to correct treatment misinformation and raise awareness of evidence-based sources of medical information [40]. Effective communication techniques between professionals and parents might facilitate the establishment of the crucial partnership required for the effective management of pediatric AD. Improved medication adherence can be achieved by teaching parents to ask questions and seek explanations when unsure about the prescribed treatment plan [41,42]. Giving patients or parents of pediatric patients thorough written instructions regarding the use of TCSs may be beneficial, as may a joint review of the instructions that addresses any queries or worries that individuals may have. When the clinic session comes to an end, patients or parents of pediatric patients can help clear up any confusion by explaining what they understood about the recommended prescription schedule [17]. A 10 to 15 min instructional session given by a dermatologist with written instructions resulted in a 43.2% drop in the fear index score in one study assessing the impact of patient education on steroid phobia [19]. Therefore, therapists should be able to identify the anxieties and concerns that their patients or parents of their pediatric patients have with TCSs with the use of the TOPICOP scale in order to tailor their conversations, target certain barriers, and create persuasive arguments to encourage patients and/or parents to follow treatment plans.

Given the strong correlation between topical corticophobia and therapy adherence in AD, topical corticophobia could be thoroughly evaluated in clinical studies as a potential outcome-influencing factor with the aid of the TOPICOP scale. However, authors from one trial assessing the TOPICOP scale as an outcome measure for reducing steroid phobia listed several concerns, such as the absence of a pre-existing gold standard [43]. Furthermore, the TOPICOP scale should be helpful in future research to assess the potential impact of patient education on topical corticophobia thresholds.

## 5. Conclusions

The results we obtained through this research showed that the Croatian version of the TOPICOP questionnaire has sufficient validity and reliability. Our findings are comparable to the results of the validation studies across other populations, which have likewise discovered the questionnaire’s excellent psychometric qualities [3,24,25,36,37,44,45,46,47,48,49]. Despite the corticophobia prevalence being comparable to other similar studies, the gender distribution does not align with previous findings that indicate a higher frequency in female individuals [23].

Even though TOPICOP was developed and validated in France, international comparative studies require additional scale validation in various countries and cultures. These international TOPICOP versions should be examined for their cross-cultural adaptability, with both an accurate translation and a posteriori verification by a medical specialist. Additionally, future investigations should work toward creating carefully monitored trials evaluating interventions that may reduce topical corticophobia and enhance AD treatment results.

## Figures and Tables

**Table 1:** Factor saturations of TOPICOP questionnaire particles ordered by factor saturations.

Questions in the Questionnaire	Factor Saturations *
I am afraid of applying too much TCS cream/ointment.	0.67
I need reassurance about TCSs.	0.67
TCSs will affect my future health.	0.62
I don’t know of any side effects, but I’m still afraid of TCSs.	0.61
TCSs can lead to asthma.	0.61
I wait as long as I can before treating myself with TCSs.	0.59
I’m afraid of putting TCS cream/ointment on certain zones, like the eyelids, where the skin is thinner.	0.59
TCSs can lead to infections.	0.55
TCSs pass into the bloodstream.	0.53
TCSs make you fat.	0.51
I stop TCS treatment as soon as I can.	0.47
TCSs damage your skin.	0.47

* The acceptance limit of factor saturations was set at 0.4.

**Table 2:** Descriptive statistics of questions on the TOPICOP questionnaire.

Questions in the Questionnaire	M(SD)
TCSs pass into the bloodstream.	2.74 (0.91)
TCSs can lead to infections.	1.85 (0.92)
TCSs make you fat.	1.92 (0.95)
TCSs damage your skin.	2.46 (0.85)
TCSs will affect my future health.	2.53 (0.81)
Topical corticosteroids will affect my health in the future.	2.06 (0.82)
I don’t know of any side effects, but I’m still afraid of TCSs.	2.58 (0.99)
I am afraid of applying too much TCS cream/ointment.	2.68 (0.94)
I’m afraid of putting TCS cream/ointment on certain zones, like the eyelids, where the skin is thinner.	2.79 (0.96)
I wait as long as I can before treating myself with TCSs.	2.30 (0.86)
I stop TCS treatment as soon as I can.	2.27 (1.12)
I need reassurance about TCSs.	2.28 (0.98)

## Data Availability

Data are contained within the article.
